# Error-prone nonhomologous end joining repair operates in human pluripotent stem cells during late G2

**DOI:** 10.18632/aging.100336

**Published:** 2011-06-12

**Authors:** Alexandra N. Bogomazova, Maria A. Lagarkova, Leyla V. Tskhovrebova, Maria V. Shutova, Sergey L. Kiselev

**Affiliations:** Stem Cell Laboratory; Vavilov Institute of General Genetics RAS; Moscow, 119991; Russia

**Keywords:** human pluripotent cells, DNA damage repair, NHEJ, chromosomal aberration, G2 chromosomal radiosensitivity assay

## Abstract

Genome stability of human embryonic stem cells (hESC) is an important issue because even minor genetic alterations can negatively impact cell functionality and safety. The incorrect repair of DNA double-stranded breaks (DSBs) is the ultimate cause of the formation of chromosomal aberrations. Using G2 radiosensitivity assay, we analyzed chromosomal aberrations in pluripotent stem cells and somatic cells. The chromatid exchange aberration rates in hESCs increased manifold 2 hours after irradiation as compared with their differentiated derivatives, but the frequency of radiation-induced chromatid breaks was similar. The rate of radiation-induced chromatid exchanges in hESCs and differentiated cells exhibited a quadratic dose response, revealing two-hit mechanism of exchange formation suggesting that a non-homologous end joining (NHEJ) repair may contribute to their formation. Inhibition of DNA-PK, a key NHEJ component, by NU7026 resulted in a significant decrease in radiation-induced chromatid exchanges in hESCs but not in somatic cells. In contrast, NU7026 treatment increased the frequency of radiation-induced breaks to a similar extent in pluripotent and somatic cells. Thus, DNA-PK dependent NHEJ efficiently participates in the elimination of radiation-induced chromatid breaks during the late G2 in both cell types and DNA-PK activity leads to a high level of misrejoining specifically in pluripotent cells.

## INTRODUCTION

Pluripotent human embryonic stem cells (hESCs) are derived from the inner cell mass (ICM) of spare blastocysts and are able to differentiate into various cell types. Therefore, these cells are often used as an in vitro model of the ICM. Recent studies suggest that a chromosomally aberrant cell population is present in nearly all human spare embryos at the cleavage stage [1-3]. However, newborns are characterized by a reduced frequency of chromosomal abnormalities when compared to preimplantation embryos [4]. In vivo, the pluripotent cell state is maintained for a very limited time; however, hESCs can be grown indefinitely in culture and their capacity to self renew and to differentiate into any cell type can be preserved for prolonged periods of time. These unique properties make hESCs very attractive as a potential source of cells for therapeutic usage. Clearly, the genome stability of hESCs is an important issue to be considered prior to use in clinical applications because even small genomic changes can significantly impair cell functionality and safety. Several reports have provided evidence of remarkable karyotype stability maintained by some hESC lines over the course of more than 140 -180 passages in vitro [5-6]. However, high-resolution karyotyping methods have established that hESCs acquire chromosomal abnormalities during long-term passaging in vitro, namely new sites of heterozygosity loss (LOH) and changes in copy-number variations (CNVs) [7, 8]. It is possible that the chromosomal aberrations observed in hESCs might reflect events similar to those that occur in a developing embryo at the blastocyst stage. Later in development, cells with normal karyotypes are selected by an unknown mechanism, but hESCs accumulate chromosomal alterations during culturing in vitro. Repair of DNA double strand breaks (DSBs) by homologous recombination (HR) could be the source of the LOH arising in hESCs during cultivation while CNVs could potentially result from DSB repair by non-allelic homologous recombination (NAHR), non-homologous end joining (NHEJ) or microhomology-mediated end joining [9, 10]. A recent study aimed at characterizing DNA repair in hESCs indicates that HR is the major, if not the sole, mechanism of DSB repair in pluripotent human cells compared to differentiated somatic cells, which typically use NHEJ [11]. However, more recently Adams et al. [12] provided evidence demonstrating NHEJ functionality in hESCs and showed that two closely-spaced DSBs induced by I-Sce endonuclease can be repaired with high fidelity by NHEJ in hESCs. NHEJ activity can result in chromosomal rearrangements when multiple DSBs coincide in space and time [13]. The aim of this study is to determine the repair accuracy of multiple radiation-induced DSBs in human pluripotent cells. To investigate the level of DSB misrejoining in pluripotent and somatic cells, we used a G2-chromosomal radiosensitivity assay [14]. We analyzed radiation-induced chromosomal aberrations in solid-stained metaphases 2 hours following irradiation, i.e., the cytogenetic analysis involved only cells irradiated during the late G2 stage of the cell cycle after transition through the G2/M checkpoint [15]. The design of this G2-assay allowed us to overcome the prominent differences in sensitivity to irradiation of pluripotent and somatic cells observed by Filion et al. [16] and their differences in cell cycle structure and regulation demonstrated by Momčilović et al. [17]. In addition, cytogenetic analysis provides a unique opportunity to estimate the frequency of misrejoining during DSB repair. We used the G2-assay to compare the accuracy of repair in pluripotent cells, isogenic somatic cells and HS27 primary fibroblasts. We show that DNA-PK-dependent NHEJ suppresses the formation of chromatid breaks after irradiation during late G2, and most of the radiation-induced chromatid exchanges observed in hESCs result from DNA-PK activity. These data elucidate the mechanisms involved in the formation of radiation-induced chromatid aberrations and propose that these mechanisms contribute to chromosome instability in pluripotent cells in vivo.

## RESULTS AND DISCUSSION

### G2-chromosomal radiosensitivity assay

The G2-chromosomal radiosensitivity assay was used to assess the chromosomal aberration frequency in cells exposed to 1 Gy of γ-irradiation and harvested 2 hours later. Two human embryonic stem cell lines (hESM01, hESKM05) had its isogenic somatic cell line: hESM01f and hESKM05f represented the fibroblast cell lines derived from their respective hESCs. Primary human foreskin fibroblasts HS27 were included in the study to compare with fibroblast cell lines derived from hESCs. For enrichment of somatic cell spectrum and additional control of possible effects of in vitro differentiation we also introduced another pair of isogenic pluripotent and somatic cells: induced pluripotent stem cells iPS12 and their parental HUVEC line.

**Figure 1:**
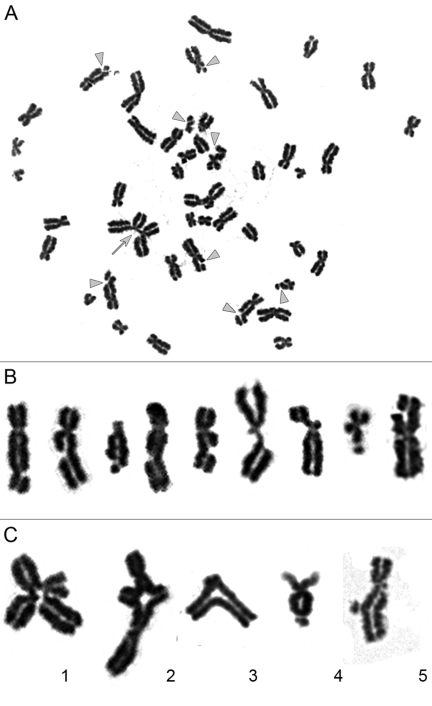
Chromatid-type aberrations observed in metaphases 2 hours after exposure to γ-irradiation. (**A**) Metaphase chromosome spread prepared from iPS12 cells irradiated at dose of 1 Gy during late G2 stage. Gray triangles indicate chromatid breaks and gray arrow indicates chromatid exchange. (**B**) Magnified images of chromosomes with chromatid breaks. (**C**) Magnified images of chromatid exchanges. **1.2** exchange of chromatid segments between different chromosomes; **3** exchange of chromatid segments between arms within chromosome; **4** exchange resulting from fusion of broken ends of chromatids within one arm of chromosome; **5** non-terminal deletions of chromatid segment with fusion of broken ends of chromatid.

Non-irradiated cell lines are characterized by low levels of chromatid fragments, with average frequencies detected ranging from 0.01-0.08 per cell (Supplemental Table S1). After irradiation at the G2 stage, we observed a highly significant increase in the frequency of chromatid-type aberrations occurring in 88-100% of the metaphases examined for each cell line. Observed chromosomal aberrations included both chromatid exchanges and numerous chromatid breaks (Figure 1). It should be noted that chromatid exchanges are the products of fusions of broken ends of chromatids (i.e., misrepair of DSBs), while chromatid breaks in the G2-assay result from the conversion of non-repaired DSBs to the visible abnormalities of chromosomes [18]. Chromatid exchanges were presented mostly by aberrations in which chromatid segments were exchanged between different chromosomes (aberrations also known as tetraradials), or between arms within chromosome. Non-terminal deletion and aberrations resulted from the fusion of broken ends of chromatids from one arm of a chromosome were also considered to be exchanges (Figure 1C). Chromatid exchanges detected in the G2-assay can theoretically give rise to translocations, pericentric inversions, rings, duplications or terminal and interstitial deletions after cytokinesis.

**Figure 2:**
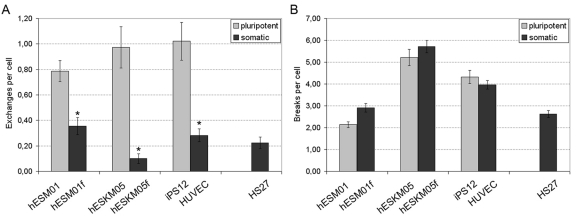
Chromatid-type aberration frequency was analyzed in pairs of isogenic pluripotent and somatic cells exposed to 1 Gy of γ-irradiation at late G2 stage. (**A**) Chromatid exchanges. (**B**) Chromatid breaks. Primary foreskin fibroblasts HS27 were used as a reference. *, significant difference was observed as compared with isogenic pluripotent cells, p < 0.0001, X^2^-test.

The exposure of pluripotent cells to γ -irradiation yields a significantly higher rate (2 - 10 fold) of chromatid exchanges when compared to matched, isogenic controls or to primary HS27 fibroblast cultures. The frequency of chromatid exchanges in pluripotent cells varied from 0.79 ± 0.08 per cell in hESM01 to 1.02 ± 0.15 per cell in iPS12. The lowest rate of exchange frequency among somatic cell lines was observed in hESKM05f (0.09 ± 0.03 per cell), while hESM01f demonstrated the highest yield of radiation-induced chromatid exchanges (0.36 ± 0.07 per cell) (Figure 2, Supplemental Table S3). Thus, highly significant differences in the frequency of radiation-induced chromatid exchanges were detected for all pairs of isogenic pluripotent and somatic cells studied (p < 0.0001). It should be noted, that virtually all chromatid exchanges observed in both cell types were readily identified as resulting from non-homologous chromosomal interaction. This observation indicates that NHEJ or non-allelic HR (NAHR) are potential sources of chromatid exchange formation. HR occurs much more slowly than NHEJ, and DSB repair by HR typically takes at least 7 hours in human fibroblasts [19]. Therefore, NHEJ appears to be the pathway responsible for the formation of chromatid exchanges observed during the 2 hours post-irradiation in our G2-assay.

The rate of radiation-induced chromatid breaks was similar in isogenic pluripotent and somatic cell lines (Figure 2B). Hence, pluripotent cells cannot be distinguished from somatic cells on the basis of the numbers of chromatid breaks in the G2-assay.

Cytogenetic analysis using the G2-assay was performed in cells irradiated during late G2 after the transition through the G2/M checkpoint. A non-functional G2/M checkpoint due to ATM deficiency or ATM inhibition can significantly increase the yield of chromatid breaks in the G2-assay [20]. Consequently, the stringency of G2/M arrest in irradiated cells was examined. To determine the stringency of G2/M arrest, we counted the number of cells entering mitosis under the same conditions used for the G2-assay by pH3 immunostaining and compared mitotic indexes in irradiated and non-irradiated cells (Supplemental Figure S2). On average, the mitotic index observed in irradiated cells corresponded to approximately 20% of the mitotic index observed in non-irradiated cells, and no significant differences in the G2/M arrest stringency were found between any of the cell lines studied. Therefore, the differences observed in the G2-assay cannot be explained by differential G2/M checkpoint characteristics.

### Dose-response of chromatid exchanges induced by irradiation in the G2 stage

**Figure 3:**
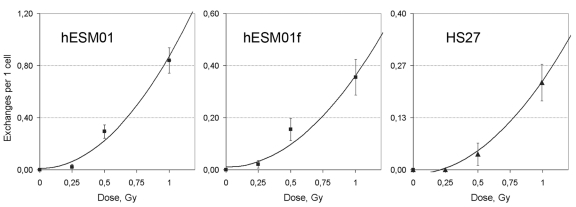
The rate of chromatid exchanges induced by γ-irradiation at G2 fitted to quadratic function in pluripotent hESM01 and differentiated hESM01f and HS27 cells.

DSB repair at the G2 stage can potentially utilize both NHEJ and HR repair pathways. The formation of chromatid exchanges between non-homologous chromosomes can be due to either from misrejoining of two DSBs in two non-homologous chromosomes by NHEJ or from processing of one DSB by NAHR. These distinct one-hit and two-hit mechanisms of chromosomal aberration formation are characterized by different types of dose-response curves. A linear curve would be expected for the one-hit mechanism because DSBs are linearly dependent from dose. However, if exchanges are formed by interaction of two DSBs, the probability of exchanges should be proportional to the square of the number of DSBs, and a quadratic curve would be expected for such two-hit events [21]. To determine the mechanisms of chromatid exchange formation in our system, we analyzed the dose-response relationship of chromatid exchanges induced by irradiation at G2 at 0.25, 0.75, and 1 Gy for hESM01, hESM01f and HS27 cell lines. The results of cytogenetic analysis are presented in Figure 3 and Supplemental Table 4.

We used a linear regression analysis to determine the type of dose-response model (linear, linear-quadratic, or quadratic) that corresponds with our data. Our calculations indicated that the rate of chromatid exchanges fit well with the quadratic function Y=b0+b1*D2, where Y is the yield of radiation-induced chromatid exchange, and D is the dose. The estimates of dose-effect coefficients are summarized in Table 1, and the curves are presented in Figure 3. The quadratic dose-response model suggests that the radiation-induced chromosomal lesions were produced by the interaction of two DSBs. Therefore, we propose that DSB misrejoining by NHEJ is the main cause of chromatid exchange formation in hESCs and differentiated cells.

**Table 1:** Curve Fitting using the Quadratic Model (Y = b_0_ + b_1_* D^2^) for Frequency of Chromatid Exchanges

Cell line	b_0_ ± SE**^a^**	b_1_ ± SE	p-level for b_1_	Adjusted R^2^
hESM01	0.01 ± 0.04	0.86 ± 0.07	0.01	0.98
hESM01f	0.02 ± 0.03	0.35 ± 0.05	0.02	0.94
HS27	−0.01 ± 0.01	0.24 ± 0.01	0.003	0.99
^a^ SE is standard error				

**Figure 4:**
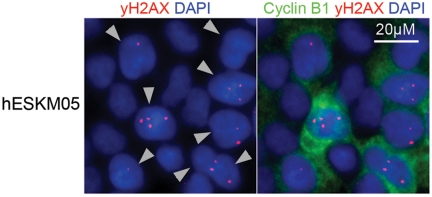
Double immunofluorescence staining of hESCs with γ-H2AX (red) and G2-specific Cyclin B1 (green) antibodies revealed high frequency of γ-H2AX foci in cells at G2 phase. Nuclei were counterstained with DAPI (blue). Gray triangles indicate G2-cells.

The dose response curve for chromosomal exchange aberrations (translocations or dicentrics) induced by γ-radiation exposure of cells at the G0/G1 phase usually includes both linear and quadratic terms: Y=b0+b1*D+b2*D2. The linear term is thought to represent an interaction of two DSBs produced along a single track, and the quadratic term represents interactions between DSBs produced independently by different tracks [21]. In our case, we observed a clear quadratic dose response of exchange yield. A similar observation of a purely quadratic dose-response relationship for chromatid exchanges in human fibroblasts has been previously described by Gotoh et al. [22]. This quadratic dose response indicates that chromatid exchanges were produced by independent radiation-induced DSBs resulting from different tracks and that no interactions between radiation-induced DSBs and endogenous DSBs occurred in pluripotent cells. It should be noted that in hESC lines we observed a high spontaneous level of γ-H2AX foci, which are well-known surrogate markers of DSBs [23]. High spontaneous levels of γ-H2AX foci have been demonstrated previously in mouse ESCs [24, 25]. Double immunofluorescence staining of hESCs with γ-H2AX and G2-specific cyclin B1 antibodies revealed cell cycle phase-related heterogeneity of γ-H2AX foci frequency, with the highest numbers of bright and clear γ-H2AX foci observed at the G2 phase (Figure 4). More specifically, the mean number of γ-H2AX foci was approximately 3 per cyclin B1-positive nucleus but less than 0.1 per cyclin B1-negative cell. However, as mentioned above, our dose-response analysis indicated that hESCs did not have endogeneous DSBs capable of interacting with radiation-induced DSBs to form chromatid exchanges during late G2. This finding indicates that there are no endogenous DSBs in hESCs during late G2 after the G2/M checkpoint transition and provides additional support to the hypothesis that most spontaneous γ-H2AX foci in ES cells are not associated with DSBs [25].

In summary, we can conclude that NHEJ is responsible for the misrejoining of DSBs in pluripotent stem cells at the late G2 after irradiation. Previously, more accurate NHEJ was observed in hESCs using an I-Sce model with only two DSB per nucleus [12]. The irradiation at a dose of 1 Gy induced approximately 40-80 DSBs per nucleus simultaneously in G2-cells [26]. This high level of DNA damage revealed an inability of hESCs to prevent DSB misrejoining by NHEJ.

### DNA-PK is important for NHEJ in hESCs during late G2

There are two NHEJ pathways characterized by different sets of factors contributing to DNA repair. Fast D-NHEJ strictly depends on DNA-PK/XRCC4/LIG4. A slow and less accurate B-NHEJ backup pathway relies on PARP1/XRCC1/LIG3 [27]. Chromosomal aberra-tions observed in the G2 assay are formed within 2 hours after irradiation, and thus, fast repair is more likely to play a role in chromatid exchange formation. Thus, to study the impact of the D-NHEJ pathway on radiation-induced chromatid aberrations in pluripotent cells, we used a competitive NU7026 inhibitor which effectively blocks DNA-PK [28]. The pluripotent cell line, hESKM05, its somatic derivative, hESKM05f, and the primary fibroblast cell line, HS27, were chosen for the experiments with chemical inhibition of NHEJ.

**Figure 5:**
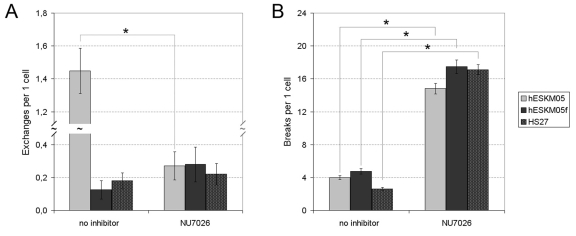
The influence of NU7026 (50 μM) on the frequency of radiation-induced chromatid-type aberrations (D = 1 Gy) in hESKM05, hESKM05f and HS27. (**A**) NU7026 decreased radiation-induced chromatid exchanges to no-zero level in hESCs and had no effect on the level of exchanges in somatic cells. (**B**) NU7026 treatment resulted in significant increase of radiation-induced chromatid breaks in all cells studied. *, yield of chromosomal aberrations significantly differs from values observed in same cells non-treated with inhibitor, X^2^- test, p < 0.0001.

The NU7026 treatment followed by 1 Gy of γ- irradiation resulted in a significant increase in radiation-induced chromatid breaks in all cell lines studied (Figure 5B). The level of chromatid breaks was elevated approximately fourfold to an average of 15-18 breaks per cell. However, radiation-induced chromatid exchanges were affected by NU7026 only in pluripotent hESKM05 cells (Figure 5A). The exchange rate in hESKM05 cells decreased by 80% (from 1.45 ± 0.10 per cell to 0.27 ± 0.09 per cell, p < 0.0001) upon DNA-PK inhibition. It should be noted that NU7026 did not cause chromosomal aberrations or additional γ-H2AX foci without irradiation (Supplemental Table S5). Immunostaining with a phospho-H3 antibody demonstrated that NU7026 treatment did not alter the number of cells that reached metaphase after irradiation, i.e. NU7026 didn't influence G2/M checkpoint (data not shown). Thus, our data indicate that DNA-PK suppresses the formation of chromatid breaks during late G2 in both types of cells studied.

DNA-PK activity was also associated with a high level of misrejoining in hESCs but not in differentiated cells. Two recent reports show that DNA-PK does not significantly contribute to DSB repair in hESCs [11, 12]. However, our data indicate that DNA-PK does contribute to DSB repair immediately after DNA damage in hESCs, at least in G2 cells after passing through the G2/M checkpoint. Future studies will be required to determine the functionality of DNA-PK dependent NHEJ during other phases of cell cycle. The genetically modified ES cells or iPS cells derived from patients with DNA repair-deficiency disorders will be especially useful in studies of DNA repair in pluripotent cells [29-32].

Laurent et al. reported recently a higher frequency of CNVs in human pluripotent stem cells compared to somatic cells [33]. Narva et al. also found that hESC cultivation led to changes in CNVs [8]. One can speculate that error-prone DNA-PK-dependent NHEJ might contribute to these genomic alterations in human pluripotent stem cells.

A high mitotic index is typical for cells of the ICM and for ES cells. The G2-assay performed in pluripotent cells revealed that exposure to radiation induces “sticky” ends of broken chromosomes at the premitotic stage in these cells. The DSBs misrejoined during G2 can give rise to a bridge-fusion-bridge cycle and to deletions and duplications of chromosome segments [34]. Complex chromosome aberrations derived from bridge-fusion-bridge cycles have been observed previously in blastomeres of cleavage embryos [3]. However, hESCs do not demonstrate any prominent genome instability, which is common for cleavage embryos. Therefore, hESCs can effectively suppress endogenous DSBs at the G2 stage, thereby decreasing the possibility of aberrant chromatid exchange or can block bridge-fusion-bridge cycles via the rapid elimination of damaged cells.

Previous work from several groups has shown that the error-free HR mechanism predominates in DSB repair in mouse and human ES cells [11, 35-36]. Recently, Adams et al. [12] provided evidence demonstrating NHEJ functionality in hESCs and showed that DSBs induced by I-Sce endonuclease can be repaired with high fidelity by NHEJ in hESCs through a DNA-PK-independent mechanism. Our data on G2-chromosomal radiosensitivity of human pluripotent stem cells also indicates on NHEJ functionality in these cells. However, we demonstrate that human pluripotent stem cells can effectively utilize a DNA-PK-dependent NHEJ mechanism for repair of radiation-induced DSBs during the late G2 stage of the cell cycle, prior to entering mitosis. Furthermore, we demonstrate that DNA-PK is responsible for the excessive misrepair of DSBs observed in hESCs compared to somatic cells.

## MATERIALS AND METHODS

### Cell cultivation

The hESC lines, hESM01 and hESKM05, were previously described by Lagarkova et al. [37]. Human umbilical vein endothelial cells (HUVECs) were derived as described in Baudin et al. [38]. The induced pluripotent stem (iPS) cell line, iPS12, was derived from HUVECs by lentiviral transfection with four transcription factors, KLF4, OCT4, SOX2 and C-MYC [39]. Fibroblast-like cells hESM01f and hESKM05f were previously established from hESM01 and hESKM05, respectively [40]. The primary foreskin fibroblast cell line, HS27, was obtained from ATCC (ATCC#CRL-1634). A summary of the immunocytochemical features of cell lines used and representative images of each are presented in Supplementary Materials (Supplemental Table S1, Supplemental Figure S1).

The pluripotent cell lines were maintained in defined medium mTeSR1 (StemCells Technologies) on Petri dishes coated with matrix Matrigel (BD). Somatic cell lines HS27, hESM01f, and hESKM05f were grown in DMEM medium supplemented with 10% fetal bovine serum (FBS, Hyclone) and 5 ng/ml hrbFGF (Peprotech), 2 mM L-glutamine, 50 units/ml penicillin and 50 μg/ml streptomycin (all from Hyclone). HUVEC were cultivated in DMEM/F12 with 15% FBS, 5 ng/ml hrbFGF (Peprotech), 20 ng/ml hrVEGF (Peprotech), 1% nonessential amino acids, 2 mM L-glutamine, 50 units/ml penicillin and 50 μg/ml streptomycin (all from Hyclone). All cell lines were maintained in 5% CO2 at 37^o^C.

### Immunocytochemistry, γH2AX foci and mitotic index counting

Cells on Petri dishes were fixed for 10 min with 4%PFA/PBS, permeabilized for 20 min with 0.1% TritonX100/PBS at room temperature and incubated for 30 min with blocking solution 2.5%BSA/PBS/0.1% Tween20. For γH2AX and cyclin B1 double staining, the cells were incubated overnight with monoclonal mouse anti-γH2AX (Upstate, 1:1000) and polyclonal rabbit anti-cyclin B1 antibodies (Santa-Cruz, 1:100) at 4oC. For mitotic index counting, the cells were incubated overnight with a polyclonal rabbit phosphorylated-Histone H3-antibody (pH3, Santa Cruz, 1:100) at 4oC. After three washing steps in PBS-0.1% Tween20, the cells were stained with Alexa Fluor 546 goat anti-rabbit IgG (Invitrogen, 1:1000) and Alexa Fluor 488 goat anti-mouse IgG (Invitrogen, 1:1000) for 1 hour at room temperature. Cell nuclei were counterstained with DAPI.

To estimate the numbers of γH2AX foci in cells at G2, the nuclei of cells with bright cyclin B1 staining were chosen. To score γH2AX foci in cyclin B1-negative cells, the nuclei of cells with cyclin B1-negative cytoplasm were selected. To estimate the frequency of γH2AX foci, 100 - 200 nuclei were scored. For mitotic index counting, the number of pH3-positive nuclei was divided by the total number of nuclei. To assess the efficiency of the G2/M checkpoint, the mitotic index was determined by scoring 3000 -5000 cells.

### Irradiation, inhibitor treatment and metaphase chromosome preparations

Cells of 70-80% confluency were irradiated with doses of 0.25, 0.5 or 1 Gy (dose rate 0.1 Gy/min) in Petri dishes at room temperature. An inhibitor of DNA-PK, NU7026 (Sigma-Aldrich), was diluted in DMSO and added to cultivating media 4 hours before irradiation at a final concentration of 50 μM. Thirty minutes after irradiation, colcemid (Invitrogene) was added at a final concentration 0.1 μg/ml. For metaphase chromosome preparations, cells were collected 120 min after irradiation. Hypotonic treatment (0.075 M KCl) was performed for 18 min at 42 oC. Cells were fixed with 2 changes of an ice-cold methanol: glacial acetic acid mix. The first fixative consisted of a mixture of methanol and glacial acetic acid at ratio 6:1, and the second fixative consisted of a methanol: glacial acetic acid mixture at a 3:1 ratio. Fixed cells were stored in fixative (3:1) at 4oC. Metaphase slides were made according to standard procedures and stained with Giemsa.

### Cytogenetic analysis

Euploid metaphases with 46 chromosomes were analyzed for the presence of chromosomal aberrations, including chromatid breaks, isochromatid breaks and chromatid exchanges. Chromatid discontinuances of lengths greater than the width of the chromatid were considered to be chromatid breaks. Chromatid discontinuances with lengths less than the chromatid width were considered to be chromatid gaps and were not counted as aberrations in the present analysis. Exchanges included chromatid interchanges between two and more chromosomes, chromatid intrachanges between arms of a chromosome, non-terminal deletion and aberrations resulted from the fusion of broken ends of chromatids from one arm of a chromosome. Examples of chromatid breaks and exchanges are presented in Figure 1.

### Statistical methods

The distribution of chromatid breaks and exchanges in each cell type was in general agreement with Poisson distribution (p>0.05, X2-test). The Poisson standard error of mean (SEM) was used as an indicator of dispersion SEM=√n/N, where “n” is the number of chromosomal abnormalities observed, and “N” is the number of metaphases scored. Statistically significant differences in the spontaneous level of chromosomal aberrations were estimated using Fisher's exact test. The significance of differences in the frequency of radiation-induced chromosomal abnormalities was estimated using Pearson's X2-test. Differences were considered statistically significant at a significance level of p <0.01. Linear regression analysis was applied to estimate the dose-response relationships of chromosomal aberration frequencies.

## SUPPLEMENTARY MATERIALS


