# Supplementary material for: Error-prone nonhomologous end joining repair operates in human pluripotent stem cells during late G2
Source: Aging (Albany NY). 2011 Jun 12;3(6):584–96. doi: 10.18632/aging.100336 (PMC3164367; doi:10.18632/aging.100336)
Supplement: Supplementary file 1 [file aging-03-584-s001.pdf]

39. Lagarkova MA, Eremeev AV, Svetlakov AV, Rubtsov NB, Kiselev SL. Human embryonic stem cell lines isolation, cultivation, and characterization. In Vitro Cell Dev Biol Anim. 2010; 46: 284-93.
40. Prokhorovich MA, Lagar'kova MA, Shilov AG, Karamysheva TV, Kiselyov SL, Rubtsov NB. Cultures of hESM human embryonic stem cells: chromosomal aberrations and karyotype stability. Bull Exp Biol Med. 2007; 144: 126-129.

SUPPLEMENTARY MATERIALS

Supplementary Table S1. Immunohistochemical features of pluripotent stem cells hESM01, hESKM05, iPS12, isogeneic fibroblast-like derivatives of hESCs, and HUVEC cells parental for iPS12. Markers of pluripotency are shown in bold, fibroblast markers – in italic.

Marker	hESM01	hESKM05	iPS12	hESM01f	hESKM05f	HUVEC
prolyl-4-hydroxylase	-	-	-	+	+	+
CD90	+	+	+	+	+	
CD105	-	-	-	+	+	+
vWF	-	-	-	-	-	+
CD31	-	-	-	-	-	+
CD30	+	+	+	-	-	-
CD44	-	-	-	+	+	+
Vimentin	+/-	+/-	+/-	+	+	+
Pan-cytokeratin	+	+	+	-	-	-
GFAP	-	-	-	-	-	-
**OCT4**	+	+	+	-	-	-
**NANOG**	+	+	+	-	-	-
**SSEA-4**	+	+	+	-	-	-
**SSEA-3**	+	+	+	-	-	-
**Tra-1-60**	+	+	+	-	-	

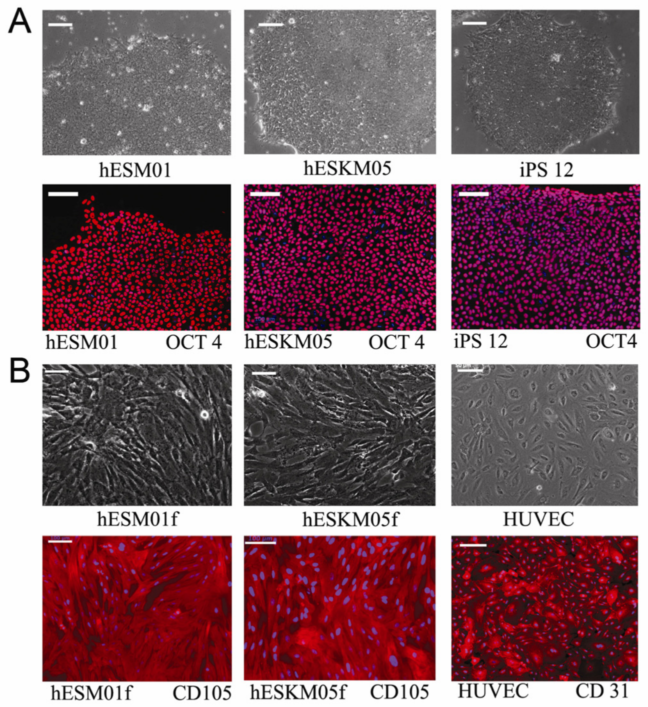

Supplementary Figure S1. The representative images of cells used in the study. (**A**) Images of pluripotent cells hESM01, hESKM05 and iPS12 cells. Upper row: Phase contrast images of cells. Bottom row: Immunofluorescence staining with antibodies to OCT4 (red) Nuclei were counterstained with DAPI (blue); (**B**) Images of differentiated hESM01f, hESKM05f and HUVEC. Upper row: Phase contrast images of cells. Bottom row: Immunofluorescence staining with antibodies to CD105 or CD31 (red). Nuclei were counterstained with DAPI (blue); Scale bars correspond to 100 µm.

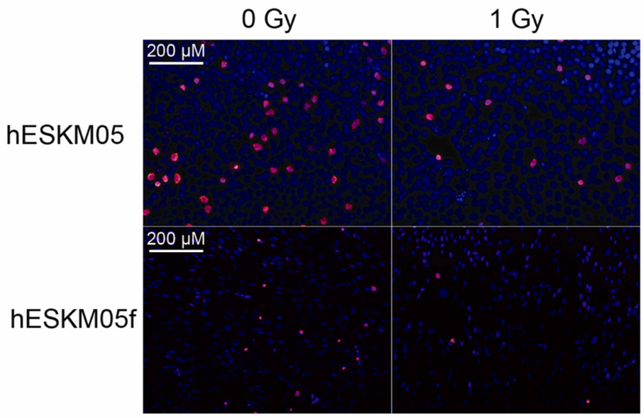

Supplementary Figure S2. The mitotic index reduced after irradiation at dose of 1 Gy to the same extent in pluripotent and somatic cells. Immunofluorescence staining with pH3-antibody (red) and DAPI (blue) counterstaining was performed.

Supplementary Table S2. The spontaneous level of chromatid-type aberration

Cell line	Cells scored	The frequency of chromatid-type aberrations per 1 cell, ± SEM[a]	
		Exchanges	Breaks
hESM01	350	0	0.01 ± 0.005
hESM01f	250	0	0.08 ± 0.02
hESKM05	60	0	0.05 ± 0.03
hESKM05f	100	0	0.04 ± 0.02
iPS12	155	0	0.01
HUVEC	100	0	0.01
HS27	50	0	0.02

[a] - SEM – Poisson's standard error of mean.

Supplementary Table S3. **The results of G2-assay**

Cell line	Cells scored	The frequency of chromatid-type aberrations per 1 cell, ± SEM[b]	
		Exchanges	Breaks
hESM01	117	0.79 ± 0.08	2.14 ± 0.143
hESM01f	76	0.36 ± 0.07*	2.91 ± 0.20
hESKM05	37	0.97 ± 0.16	5.22 ± 0.38
hESKM05f	78	0.09 ± 0.03*	5.65 ± 0.27
iPS12	46	1.02 ± 0.15	4.33 ± 0.31
HUVEC	106	0.28 ± 0.05*	3.96 ± 0.19
HS27	103	0.22 ± 0.05	2.62 ± 0.16

***** - yield of aberrations significantly differs from values observed in isogenic pluripotent cells, X^2- test, $p < 0.0001$

Supplementary Table S4. Dose response of chromatid-type aberrations

Cell line	Dose, Gy	Cells scored	The frequency of chromatid-type aberrations per 1 cell, ± SEM	
			Exchanges	Breaks
hESM01	0	350	0	0.01 ± 0.005
	0.25	47	0.02	0.47 ± 0.10
	0.5	109	0.29 ± 0.05	0.94 ± 0.09
	1	87	0.86 ± 0.10	1.53 ± 0.13
hESM01f	0	251	0	0.08 ± 0.02
	0.25	96	0.02 ± 0.01	0.66 ± 0.08
	0.5	84	0.15 ± 0.04	1.90 ± 0.15
	1	76	0.36 ± 0.07	2.91 ± 0.20
HS27	0	50	0	0.02
	0.25	50	0	0.74 ± 0.12
	0.5	50	0.04 ± 0.03	1.48 ± 0.17
	1	103	0.22 ± 0.12	2.62 ± 0.32

Supplementary Table S5. The effect of NU7026 treatment on non-irradiated cells

Cells	Inhibitor	Cells scored	The frequency of chromatid-type aberrations per 1 cell, ± SEM	
			Exchanges	Breaks
hESKM05	no inhibitor	60	0	0.05 ± 0.03
	NU7026	50	0	0.02
hESKM05f	no inhibitor	100	0	0.04 ± 0.02
	NU7026	50	0	0.02
HS27	no inhibitor	50	0	0.02
	NU7026	50	0	0

Supplementary Table S6. The results of G2-assay performed upon NU7026 treatment

Cells	Inhibitor	Cells scored	The frequency of chromatid-type aberrations per 1 cell, ± SEM	
			Exchanges	Breaks
hESKM05	no inhibitor	76	1.45 ± 0.14	4.00 ± 0.23
	NU7026	37	0.27 ± 0.09*	14.81 ± 0.63*
hESKM05f	no inhibitor	40	0.13 ± 0.06	4.75 ± 0.34
	NU7026	25	0.28 ± 0.11	17.48 ± 0.84*
HS27	no inhibitor	78	0.18 ± 0.05	2.63 ± 0.18
	NU7026	50	0.22 ± 0.07	17.12 ± 0.59*

* - yield of aberrations significantly differs from values observed in cells non-treated with inhibitors, X^2- test, $p < 0.0001$
